# Four hours of veno-venous extracorporeal membrane oxygenation using bi-caval cannulation affects kidney function and induces moderate lung damage in a mouse model

**DOI:** 10.1186/s40635-019-0285-7

**Published:** 2019-12-16

**Authors:** Ruslan Natanov, Abdurasul Khalikov, Faikah Gueler, Ulrich Maus, Erin C. Boyle, Axel Haverich, Christian Kühn, Nodir Madrahimov

**Affiliations:** 1 Department of Cardiothoracic, Transplantation and Vascular Surgery, Hannover Medical School, Hannover, Germany; 2 Department of Nephrology, Hannover Medical School, Hannover, Germany; 3 Department of Pneumology, Hannover Medical School, Hannover, Germany; 4 Clinic of Cardiothoracic Surgery, University Clinic of Würzburg, Oberdürrbacher Strasse 6, 97080 Würzburg, Germany

**Keywords:** Mouse model, Extracorporeal membrane oxygenation, Single cannula, Double lumen cannula

## Abstract

**Background:**

Improvement of single site cannulation for extracorporeal membrane oxygenation (ECMO) therapy is pivotal for reduction of patient morbidity and mortality in respiratory failure. To further improve the cardiopulmonary outcomes and reduce end organ damage, we established a murine model for single site cannulation with a double lumen cannula.

**Results:**

We created a hemodynamically stable double lumen cannula and successfully implanted it through the jugular vein into the upper and lower vena cava. This allowed adequate drainage of the blood. Blood gas analysis showed excellent oxygenation and CO_2_ reduction. There was no excessive bleeding. No signs of right heart congestion were present which was confirmed in the histological analysis of the liver. Histology demonstrated moderate lung damage and mild acute kidney injury. Neutrophil infiltration was similar in ECMO and sham kidneys.

**Conclusions:**

Veno-venous extracorporeal circulation deteriorates kidney function and promotes moderate pulmonary damage.

## Introduction

Extracorporeal membrane oxygenation (ECMO) is an essential tool in cardiorespiratory failure [1]. In isolated respiratory failure, veno-venous (vv)-ECMO is the therapy of choice as bridge to either recovery or transplantation [2]. In this setting, there are two cannulas placed in the veins of the patient. The draining cannula is typically placed through the femoral vein and advanced via the inferior vena cava to the right atrium. The returning cannula is typically placed in the right jugular vein and advanced to the border of the right atrium and upper vena cava. Through the membrane oxygenator, blood is supplied with oxygen and carbon dioxide is washed out. Indications for vv-ECMO implementation include severe acute respiratory distress syndrome (ARDS), exacerbation of chronic obstructive pulmonary disease (COPD), and as a bridge to lung transplantation [3, 4]. Although this system is highly effective, complications including bleeding, pneumothorax, and cannula infections have been reported during cannulation and ongoing ECMO therapy [2, 4, 5]. Moreover, patients on awake ECMO are difficult to mobilize when the femoral vein is cannulated. The use of a double lumen cannula has been proposed to optimize physiotherapy, patient mobilization, and patient comfort [6–8]. Additionally, placement of a double lumen cannula at a single site is less traumatic and has been proven to be very effective when CO_2_ elimination is needed. Major limitations of this system include reduced venous backflow [9], relatively low blood flow [10], cannula displacement, and thrombosis [11].

To further improve patient outcomes and reduce morbidity, a reliable and reproducible animal model is needed for research purposes. Based on our previously established mouse model of vv-ECMO [12], here we describe a novel murine double lumen cannula for vv-ECMO support.

## Material and methods

### Animals

Nineteen male C57Bl/6 mice were obtained from Charles River (Sulzfeld, Germany) and used for the experiments. Animals were randomly divided into sham-operated (*n* = 6) animals, 4 h vv-ECMO (*n* = 8) animals, and animals used in the design of the double lumen cannula (*n* = 5, see below). The weight of mice ranged between 25 and 35 g. This study was performed in compliance with the German Animal Protection Law (TierSchG) and was approved by the local animal welfare committee (Lower Saxony State Office for Consumer Protection and Food Safety, Protocol TSA 33.12-42502-04-16/2250).

### Design and construction of the double lumen cannula

**Fig. 1:**
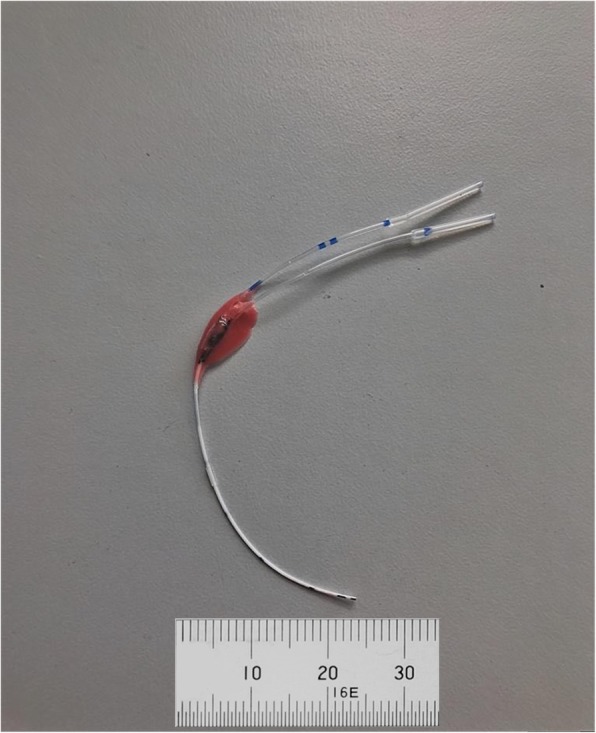
The two French double lumen cannula which were the basis for the double lumen single ECMO cannula. Ruler for reference in mm

**Fig. 2:**
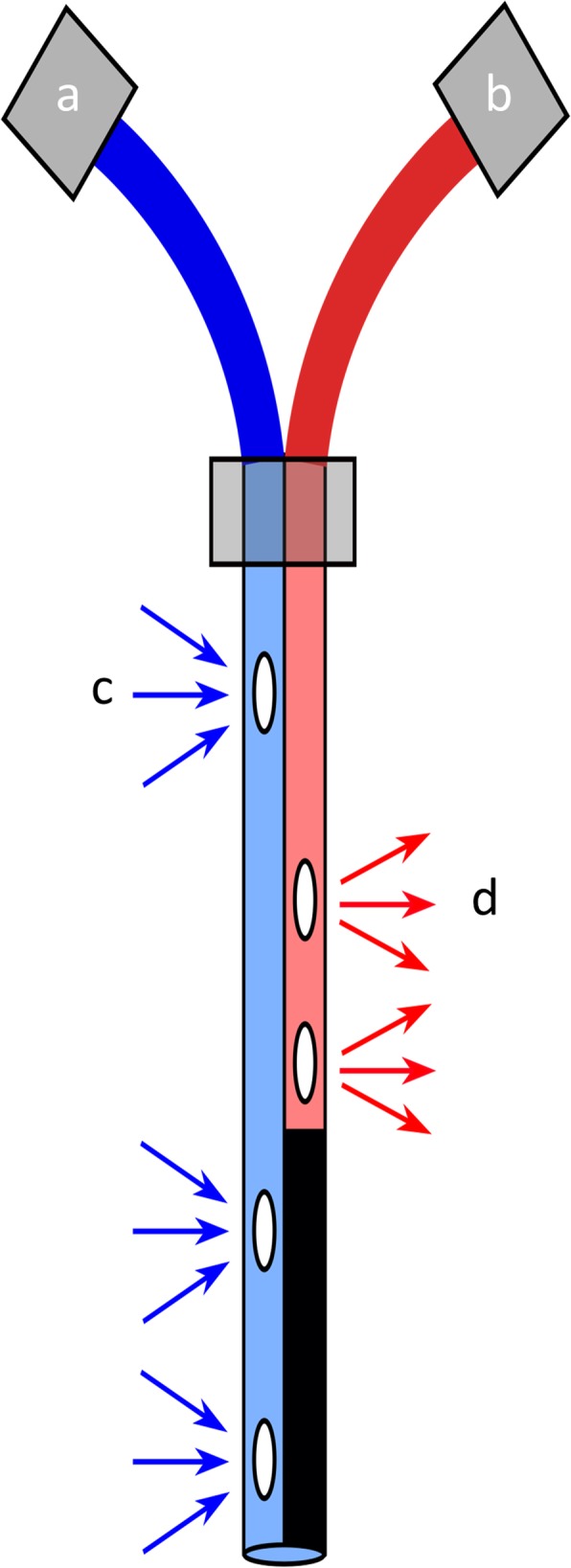
Schematic depiction of the double lumen silicone cannula. Two lines were constructed into a single cannula. One line extracts blood (**a**) from the upper and lower vena cava before passing it through the oxygenator and giving the blood back in the right atrium (**b**). Blue (**c**) and red arrows (**d**) show the fenestrations in the cannula

A 2F double lumen silicone-based catheter (Vygon GmbH & CO.KG Medizintechnik, Aachen, Germany) was used as the basis for the production of the double lumen cannula (Fig. 1). Using a sharp blade, outflow fenestrations were made in the catheter at the eventual site of the superior vena cava and inferior vena cava (Fig. 2). Similarly, inflow fenestrations were made at the height of the right atrium. To ensure optimal positioning of the fenestrations, multiple measurements were performed on five mouse cadavers of a similar age/size to the experimental mice. For optimal drainage and minimal shunting, venous outflow fenestrations were made 0.2 mm, 0.4 mm, and 20 mm from the distal end. Atrial inflow fenestrations were made 0.4 mm and 0.5 mm from the distal end. To prevent recirculation and shunting of the blood, the distal end of the inflow cannula lumen was sealed.

### Surgical procedure and extracorporeal membrane oxygenation

**Fig. 3:**
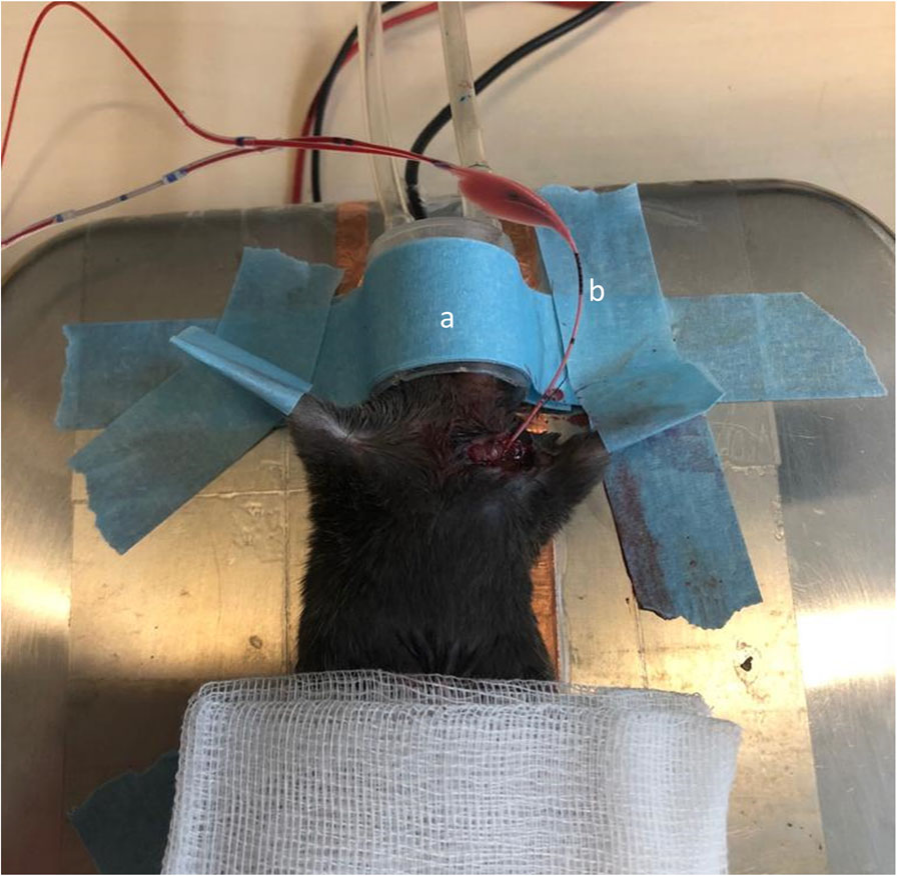
Experimental setup with mask narcosis (**a**) and double lumen cannulation through the jugular vein (**b**). During vv-ECMO, blood was circulated via a self-made oxygenator and returned via the right atrium

Surgical preparation (Fig. 3) and ECMO setup were carried out as previously described [12]. In brief, all animals were anesthetized with isoflurane mask narcosis (Fig. 3 (a)) and spontaneous breathing was maintained. Subcutaneous carprofen (Zoetis, Parsippany, NJ, USA) injections were given as additional analgesia (5 mg/kg body weight). Perfusion solution consisted of a 1:1 solution of Tetraspan: Sterofundin (B Braun Medical, Melsungen, Hesse, Germany) that had been heparinized (30 IU/ml). Prior to cannulation, the ECMO circuit was primed with 500 μl of perfusion solution. Buffering of the solution was carried out using 2.5% v/v of an 8.4% solution of sodium bicarbonate. An arterial pressure line in the left femoral artery was used for blood sampling and blood pressure monitoring.

For cannulation, a lateral skin incision on the left side of the neck was made to expose the left jugular vein. An 8-0 silk suture was placed cranially to ligate the distal segment, and a slip knot was placed at the proximal end of the vein. After introducing the double lumen cannula into the left jugular vein (Fig. 3 (b)), it was moved 3.5 cm in the direction of the superior vena cava, and further into the inferior vena cava. The cannula was then secured using slip-knots. After confirmation of correct position of the cannula, extracorporeal circulation was started and continued for 4 h. In the sham group, the surgical procedure for cannulation was identical to the ECMO animals; however, no extracorporeal circulation was commenced. In both ECMO- and sham-treated animals, the procedure was performed for 4 h.

### Blood gas analysis

Blood gas analysis (BGA) was performed from blood sampled from the femoral artery to evaluate the oxygenation and metabolic state of animals undergoing vv-ECMO.

### Blood tests of kidney and liver function

Blood samples were collected via the femoral artery prior to the procedure and via exsanguination after termination of the experiment. Serum was stored at − 20 °C for later analysis. Clinical chemistry was done using an Olympus analyzer (AU 400) according to the manufacturer’s instructions to evaluate liver enzymes (glutamate oxaloacetate transaminase (GOT) and glutamate pyruvate transaminase (GPT)) and kidney function (creatinine, urea) after vv-ECMO. All values were corrected for hemodilution as previously described [13]. Furthermore, to evaluate hemolysis during the experiment, lactate dehydrogenase (LDH) values were compared between the start of the experiment and after 4 hours.

### Histology

For organ fixation, lungs were filled with 4% paraformaldehyde via injection into the trachea. Filled lungs were explanted and incubated in 4% formalin overnight at 4 °C. After dehydration and deparaffinization, lungs were stained with hematoxylin and eosin (H&E) and histologically assessed for pulmonary damage. The liver and kidney were also collected, fixed in 4% paraformaldehyde, and stored for 24 h at 4 °C. Two-micrometer paraffin sections were stained with periodic acid-Schiff (PAS) to evaluate kidney and liver morphology. Assessment of acute kidney injury (AKI) and liver damage was done using a method previously described [13].

### Statistics

Statistical analyses were performed using GraphPad Prism version 5.0 software (GraphPad Software Inc., San Diego, CA, USA). The Kolmogorov-Smirnov test revealed the data was normally distributed. One-way ANOVA with post hoc Bonferroni tests were used for statistical analysis. Unless otherwise stated, data are presented as mean ± standard deviation (SD).

## Results

### Hemodynamics and oxygenation after 4-h sham and ECMO treatment

**Fig. 4:**
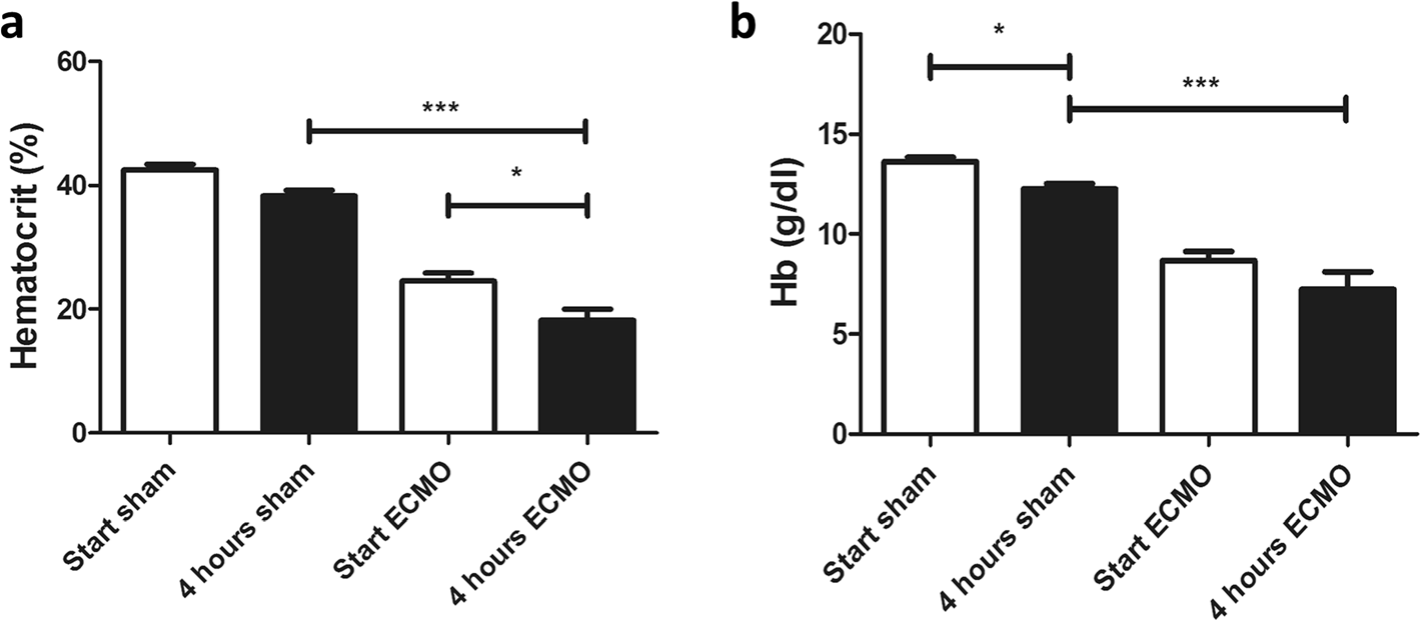
Hematocrit (**a**) and hemoglobin (**b**) values of sham-operated (*n* = 6) and 4-hour ECMO-treated (*n* = 8) animals significantly decreased over the course of the experiment. All values are given as mean ± SD.*p < 0.05; **p < 0.01, ***p < 0.0001, ns not significant

During the experiments, stable hemodynamics were observed. No excessive blood loss was noted, and this was confirmed by the relatively stable hematocrit (Hct) and hemoglobin (Hb) values. Sham-operated animals had an initial Hct value of 42.5 ± 0.9%, which dropped after 4 h to 38.3 ± 0.9% (not significant, n.s.) due to regular blood samplings (Fig. 4a). In general, approximately 150 μl of blood was taken over the course of the experiment to analyze BGA in both arterial and venous blood. There was a similar drop in Hct seen in the vv-ECMO-treated group with an initial value of 24.5 ± 1.3% and an end value of 18.3 ± 1.7% (*p* = 0.027) (Fig. 4a). A significantly lower hematocrit was measured after 4 h of ECMO in comparison to 4 h sham (*p* = 0.0016) (Fig. 4a). These findings were comparable to our previous data [14], and the lower values were due to hemodilution.

Hemoglobin showed a similar drop over the course of the experiments. The initial level of Hb of 13.6 ± 0.2 g/dL in the sham group was in the normal range and significantly dropped during the experiment to 12.2 ± 0.3 g/dL (*p* = 0.003) due to blood sampling (Fig. 4b). Although not statistically significant, a fall in hemoglobin from 8.7 ± 0.4 to 7.2 ± 0.4 g/dL was observed in the ECMO treatment group (*p* = 0.059), probably due to hemolysis caused by the ECMO.

**Fig. 5:**
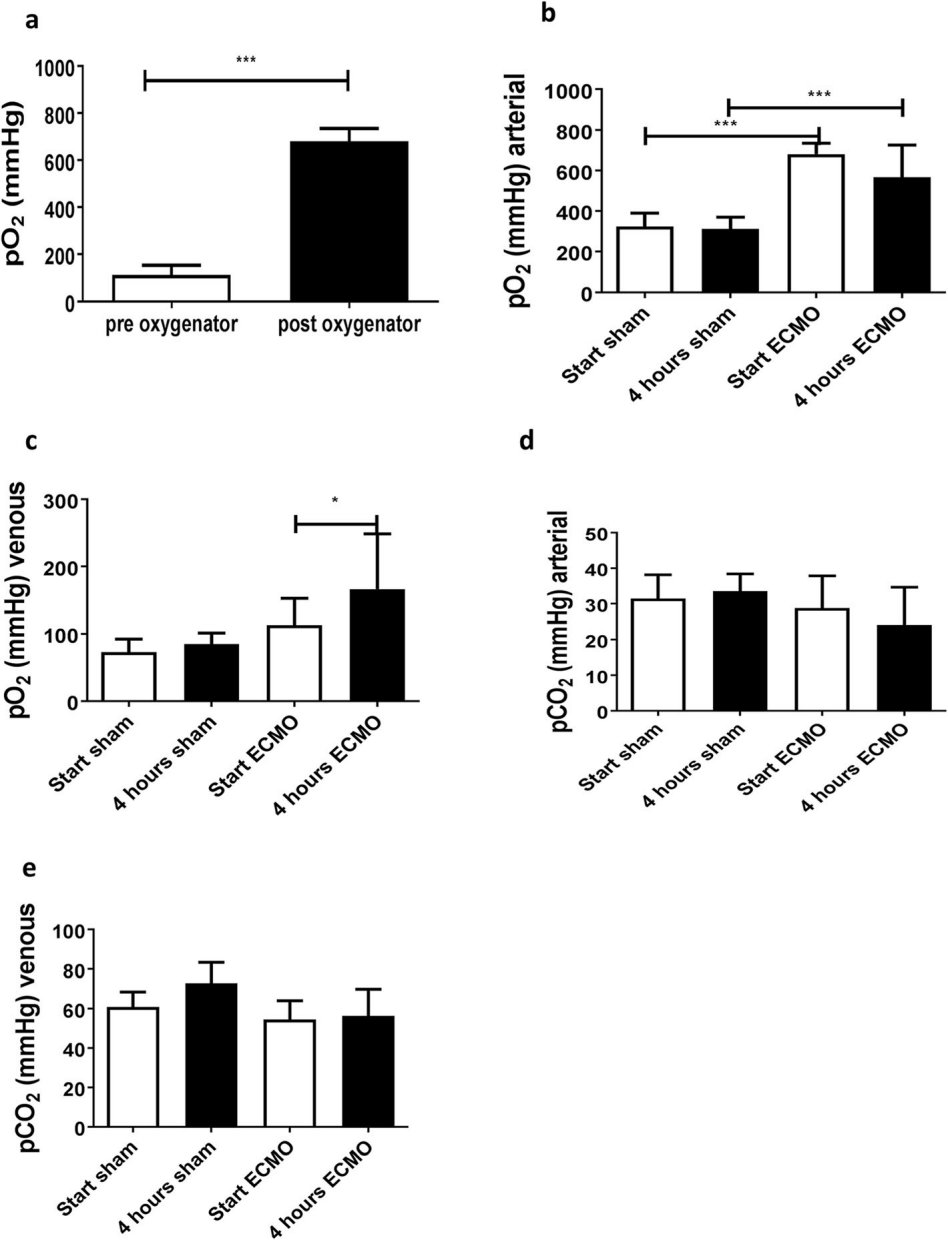
Blood gas analysis (BGA) was performed to ensure proper function of the oxygenator (**a**). pO_2_ (**b**, **c**) and pCO_2_ (**d**, **e**) values were evaluated in the ECMO animals (*n* = 8) and sham animals (*n* = 6). All values are given as mean ± SD.*p < 0.05; **p < 0.01, ***p < 0.0001, ns not significant

To ensure proper membrane oxygenator function, blood sampling was performed directly before and after the oxygenator following initiation of vv-ECMO. Pre-oxygenator pO_2_ (113.0 ± 14.1 mmHg) and post-oxygenator pO_2_ (680.9 ± 19.2 mmHg) showed a significant increase (*p* < 0.0001) indicating an excellent oxygenation capacity of our membrane oxygenator (Fig. 5a). To evaluate animal oxygenation, arterial BGAs were taken at the beginning of the experiment and were compared to BGAs taken after 4 h. Once initiated, ECMO was associated with high pO2 levels, which did not significantly change during the 4-h course of ECMO (*p* = 0.09) (Fig. 5b). After 4 h, the ECMO group showed a significantly higher arterial pO_2_ (567.1 ± 56.1 mmHg) (*p* < 0.001) compared to the sham group (312.0 ± 23.4 mmHg) (Fig. 5b). No significant differences in arterial pCO2 were recorded between the start and end of the experiment in the sham or ECMO groups (Fig. 5b). Similarly, no significant differences in venous pH or sO_2_ were observed between the start and end of the experiment for sham- or ECMO-treated animals (data not shown).

Venous blood taken from the inferior vena cava showed better pO_2_ values after 4-h ECMO therapy (*p* = 0.036) compared to the sham animals (Fig. 5c). There was no significant rise in venous blood pO_2_ after 4-h ECMO compared to start of the experiment (*p* = 0.17). No significant decrease in pCO_2_ values was seen over the course of 4 h in either sham- or ECMO-treated animals (Fig. 5d, e).

### Evaluation of organ damage after 4-h sham or ECMO treatment

**Fig. 6:**
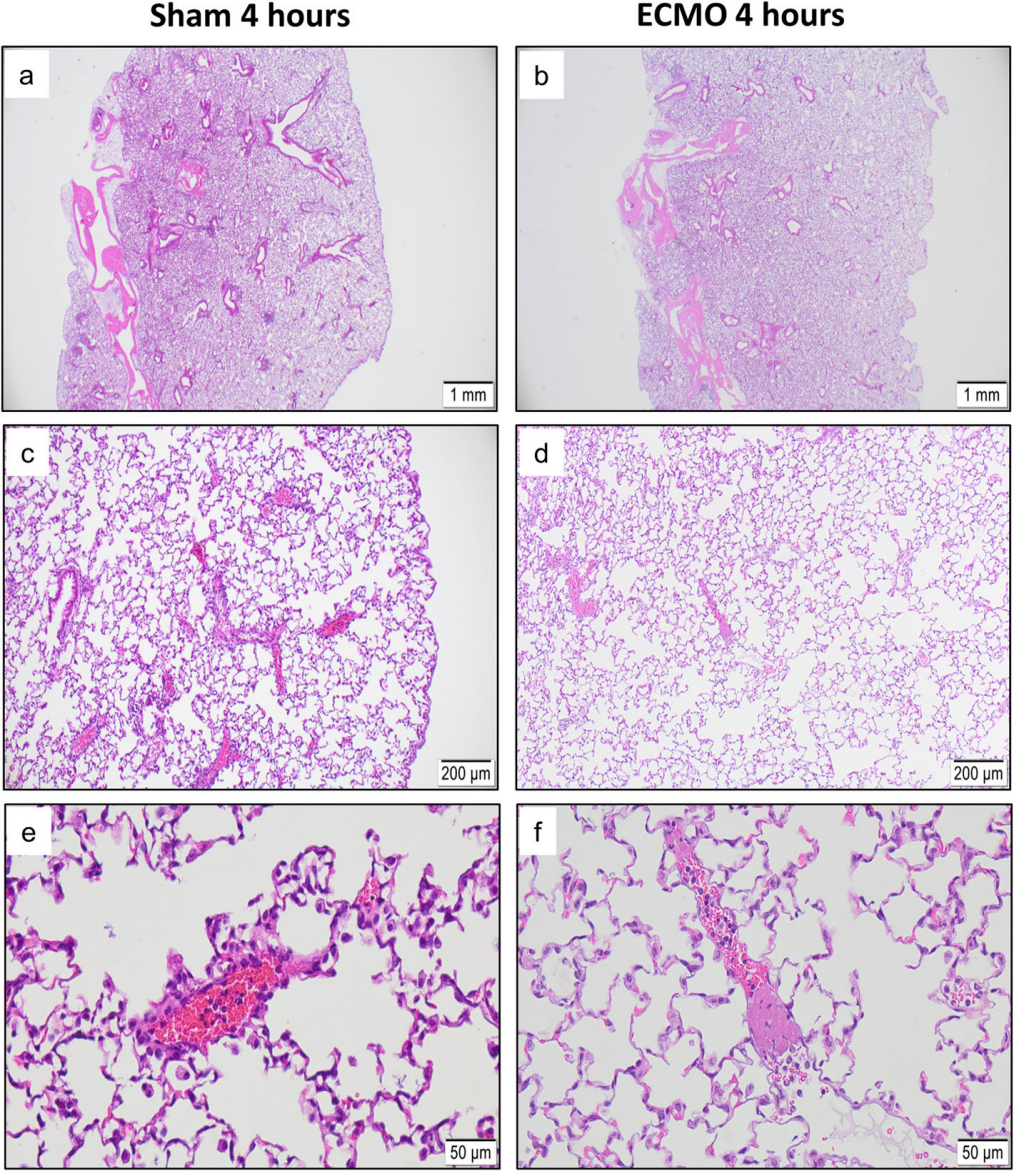
Effect of ECMO treatment on lung histology. Shown are representative images of animals in either the sham (**a**, **c**, **e**) or ECMO oxygenation group (**b**, **d**, **f**) at the end of the procedure. Both groups showed normal bronchial and vascular architecture and normal general lung histology. Interestingly, animals treated with 4-h ECMO oxygenation frequently showed peripheral small vessel coagulopathy. Original magnifications × 2 (**a**, **b**), × 10 (**c**, **d**), and × 40 (**e**, **f**)

Four hours after either sham operation or vv-ECMO with the double lumen single cannulation technique, organ damage was evaluated. No pulmonary pathology was seen after 4 h in the sham group (Fig. 6a, c, e). Although the ECMO-treated animals showed regular bronchial and vascular architecture, small peripheral vessel coagulopathy was more frequently observed compared to sham-operated animals (Fig. 6).

**Fig. 7:**
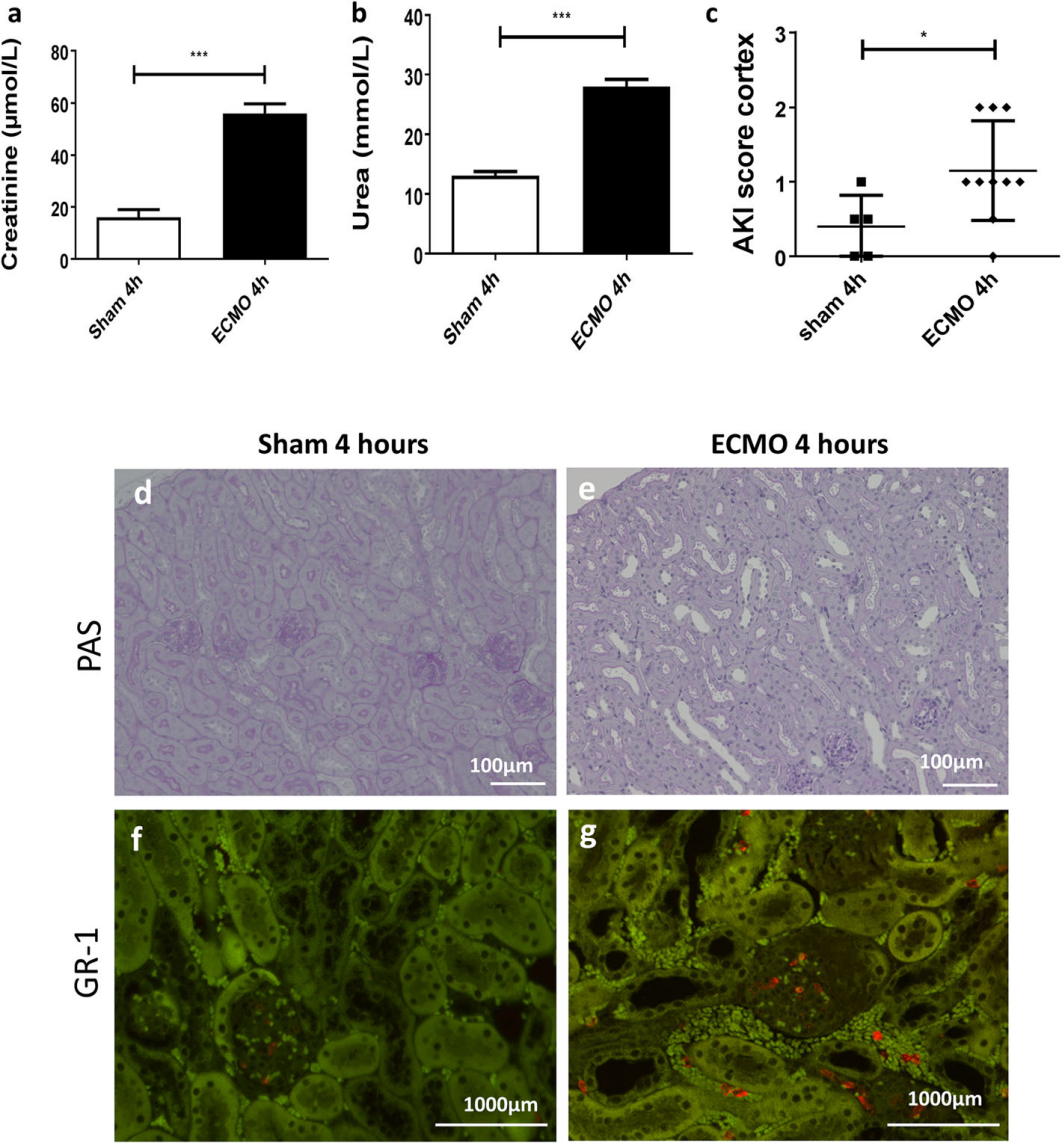
Renal function and kidney histology. Clinical chemistry showed significantly elevated serum creatinine and urea in the ECMO group (**a**, **b**). Quantification of kidney damage after PAS stain was done by AKI score which showed mild AKI with distended tubuli in the ECMO group and almost normal renal morphology in sham kidneys (**c**, **d**, **g**). GR-1 neutrophil infiltration slightly increased after ECMO in glomeruli and in the tubulo interstitial space (**e**, **f**, **h**). All values are given as mean ± SD.*p < 0.05; **p < 0.01, ***p < 0.001, ns not significant (bar represents 100 μm)

Next, renal function and kidney histology were assessed. Significant differences in serum creatinine and urea (*p* < 0.001) were seen after ECMO treatment compared to sham-operated animals (Fig. 7a, b). These differences may be due to either pre-renal acute kidney injury or hemolysis. Quantitative scoring (Fig. 7c) and histological evaluation (Fig. 7d, e) of the kidney showed a significant increase in the AKI (*p* = 0.040) score in the ECMO group, suggesting decreased renal function after vv-ECMO. Furthermore, although more GR-1 staining due to neutrophil infiltration was seen in the kidney after 4-h ECMO compared to sham-treated animals (Fig. 7f, g), quantitative scoring did not reach significance (*p* = 0.845).

**Fig. 8:**
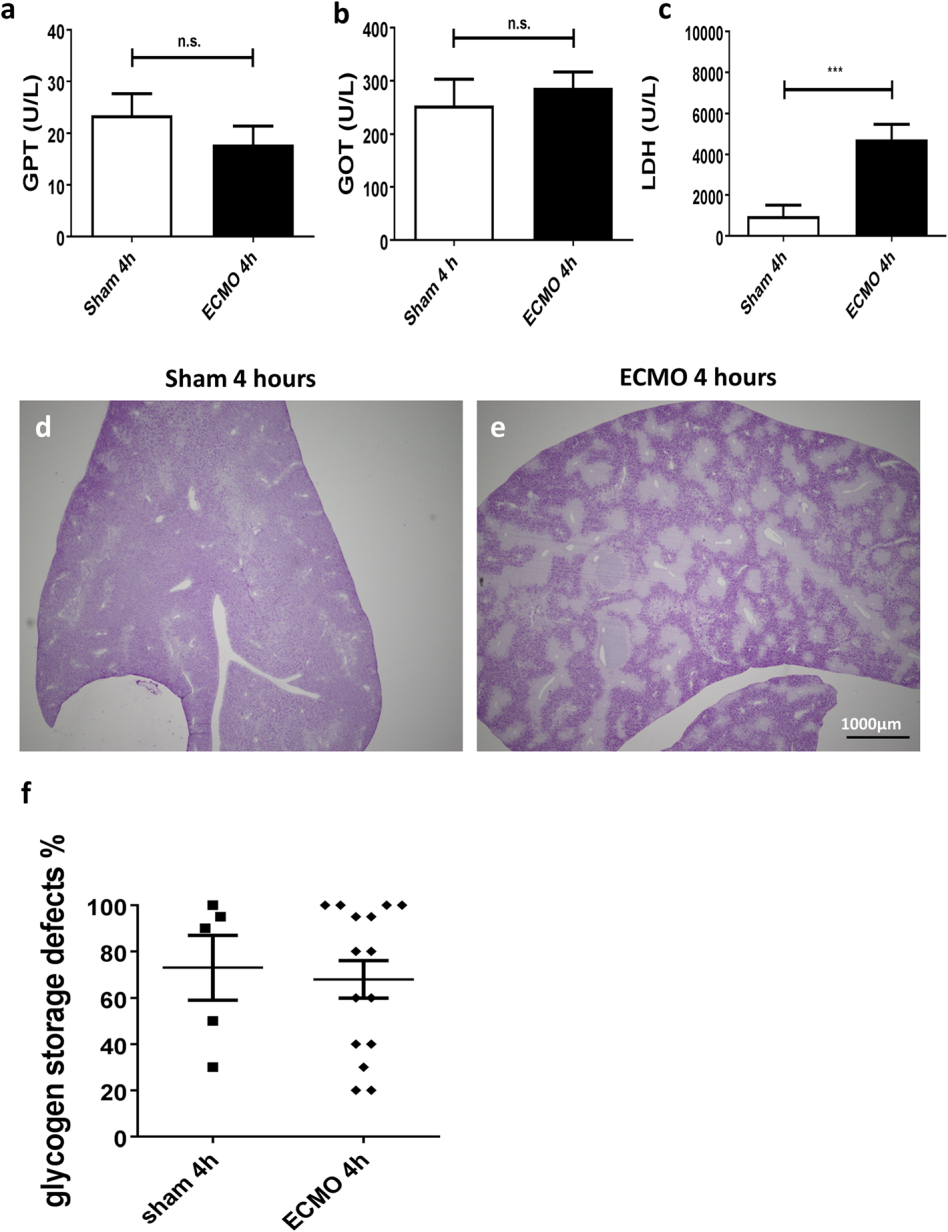
Clinical chemistry and histology of the liver. Blood samples taken after 4 h showed no significant elevation in GOT and GPT in the ECMO animals when compared to sham animals (**a**, **b**). LDH was significantly elevated in the ECMO treatment group compared to the sham group (**c**). Although histological evaluation of the liver did show reduced glycogen storage capacity in the ECMO group (**d**, **e**) compared to the sham group, this was not statistically significant (**f**). All values are given as mean ± SD.*p < 0.05; **p < 0.01, ***p < 0.0001, ns not significant (bar represents 1000 μm)

After 4 h, blood tests for liver function revealed no significant differences between ECMO- and sham-treated animals (Fig. 8a, b). Congestive hepatopathy can occur upon congestive heart failure, but this data suggests right heart congestion did not occur during the procedure. No histological abnormalities were seen in the H&E- and PAS-stained cardiac samples (data not shown). As another measure of tissue damage, LDH was measured in the blood. After 4 h, a significant increase in LDH concentration was seen in ECMO-treated animals compared to sham treatment (*p* < 0.001) (Fig. 8c).

Liver pathology was evaluated using PAS staining and showed various extents of irregular glycogen loss in all animals (Fig. 8d, e). However, there were no signs of necrosis, cellular infiltration, or edema in liver tissue. Furthermore, quantification of glycogen storage defects (Fig. 8f) did not show a significant difference in either treatment group (*p* = 0.75).

## Discussion

Animal models of bi-caval ECMO are rare, with to date, only two swine models being described [15, 16]. To our knowledge, no papers on bi-caval double lumen ECMO in a murine model have been published, making this a novel model. Although the classic two cannula vv-ECMO has become increasingly popular in patients with respiratory failure, this modality has its limitations. In small pediatric patients, there is no possibility for a second cannula as the femoral veins are often too small for cannulation. As previously stated, patient immobilization due to ECMO makes physical therapy practically impossible and may prolong the intensive care unit stay. In awake ECMO patients, ECMO tolerability is much greater and was not associated with an increase in complications such as bleeding [8, 10]. For this group of patients, a bi-caval double lumen cannula has been proposed as a therapeutic option [17]. This single cannula is inserted via the jugular vein and is ideally positioned using transesophageal echocardiography [18]. The main objective of vv-ECMO is carbon dioxide (CO_2_) elimination and, in part, supportive oxygenation in respiratory failure. Depending on the blood flow and diameter of the cannula, the rate of CO_2_ elimination can be regulated. Our new small animal model of double lumen ECMO is a proof of concept of the technique for potential future use to explore the pathomechanisms of SIRS, respiratory compensation in acute or end-stage lung disorders, and other systemic complications purely related to the blood damage caused by extracorporeal circulation. Additionally, there are commercially available genetically modified mice that develop, for example, respiratory insufficiency directly after birth or lung fibrosis in older age, in which ECMO could be studied in the context of these underlying diseases. Moreover, our cannulation technique can be successfully used in other experimental models involving dialysis, cytokine adsorbtion, plasmapheresis, or hemadsorbtion due to a similarity in function with other venous double lumen catheters widely used in the clinic.

Our results showed a feasible, hemodynamically stable setup with proper oxygenation and a trend of decreasing CO_2_ values. This suggests an optimized functioning of the oxygenator and a minimal shunting of the ECMO blood. No clinical signs of heart congestion were seen. Furthermore, clinical chemistry showed no increase in GOT and GPT values, thus making right heart congestion less probable. Increases in creatinine and urea values suggest a decrease in kidney function. Simultaneously, a significant increase in LDH was seen which may be explained due to hemolysis during ECMO. Hemolysis has previously been associated with impaired acute kidney failure after cardiopulmonary bypass [19]. Kidney failure during extracorporeal circulation is common and has multiple causes. In our model, the AKI was apparent with tubular damage upon histological assessment. Previous work showed an association between tubular damage and hemolysis, reduced renal blood flow, and hypoxia in ECMO [20–24]. AKI is an expected clinically relevant consequence of ECMO, and therefore, our model can be used to explore therapeutic interventions to prevent organ damage. In addition, due to the availability of a large number of genetically modified mouse strains, the model could also be used to investigate the molecular mechanisms leading to ECMO-related organ damage. As it is often difficult to distinguish whether complications seen clinically are caused by ECMO itself or arise from the underlying disorders accompanying acute and end-stage diseases, our model will allow researchers to specifically study the effects of ECMO on a healthy organism.

Our model did not show a significant drop in CO_2_ concentration in the arterial BGA over the course of a 4-h ECMO procedure. We believe this was due to spontaneous breathing of the animals. However, a clear trend in CO_2_ reduction was observed. A further reduction in CO_2_ values may be achieved by increasing gas flow or EMCO blood flow. Inducing a hypercapnic state in a mouse model would be beneficial to address this issue. As there are no small animal models available, we could not compare these findings. Large animal ECMO models, however, showed similar oxygenation results and comparable hemodynamics but did not report renal or hepatic function parameters [15, 16]. In conclusion, we present the proof-of-concept use of bi-caval double lumen ECMO in a murine model. Future projects include application of this model in a murine lung disease model to determine the outcome using different ECMO modalities.

## Conclusion

Veno-venous extracorporeal membrane oxygenation elicits a decrease in renal function and significantly more histological renal damage. Furthermore, it induces pulmonary morphological changes after 4 h in a murine model. Liver function and histology does not seem to be affected, neither were there signs of right heart congestion.

## Data Availability

The datasets used or analyzed during the current study are available from the corresponding author on reasonable request.

## Abbreviations

AKI: Acute kidney injury
ARDS: Acute respiratory distress syndrome
BGA: Blood gas analysis
COPD: Chronic obstructive pulmonary disease
ECMO: Extracorporeal membrane oxygenation
GOT: Glutamic oxaloacetic transaminase
GPT: Glutamate pyruvate transaminase
Hb: Hemoglobin
Hct: Hematocrit
LDH: Lactate dehydrogenase
n.s.: Not significant
PAS: Periodic acid-Schiff
SD: Standard deviation
vv: Veno-venous
